# Pharmacological Activation/Inhibition of the Cannabinoid System Affects Alcohol Withdrawal-Induced Neuronal Hypersensitivity to Excitotoxic Insults

**DOI:** 10.1371/journal.pone.0023690

**Published:** 2011-08-19

**Authors:** Marina Rubio, Hélène Villain, Fabian Docagne, Benoit D. Roussel, José Antonio Ramos, Denis Vivien, Javier Fernandez-Ruiz, Carine Ali

**Affiliations:** 1 INSERM U919 Serine Protease and Pathophysiology of the Neurovascular Unit, UMR CNRS 6232 CINAPS, Caen, France; 2 Departamento de Bioquímica y Biología Molecular, Instituto Universitario de Investigación en Neuroquímica, Universidad Complutense, Madrid, Spain; 3 Centro de Investigación Biomédica en Red sobre Enfermedades Neurodegenerativas (CIBERNED), Universidad Complutense, Madrid, Spain; 4 Instituto Ramón y Cajal de Investigación Sanitaria (IRYCIS), Facultad de Medicina, Universidad Complutense, Madrid, Spain; National Institute on Aging Intramural Research Program, United States of America

## Abstract

Cessation of chronic ethanol consumption can increase the sensitivity of the brain to excitotoxic damages. Cannabinoids have been proposed as neuroprotectants in different models of neuronal injury, but their effect have never been investigated in a context of excitotoxicity after alcohol cessation. Here we examined the effects of the pharmacological activation/inhibition of the endocannabinoid system in an in vitro model of chronic ethanol exposure and withdrawal followed by an excitotoxic challenge. Ethanol withdrawal increased N-methyl-D-aspartate (NMDA)-evoked neuronal death, probably by altering the ratio between GluN2A and GluN2B NMDA receptor subunits. The stimulation of the endocannabinoid system with the cannabinoid agonist HU-210 decreased NMDA-induced neuronal death exclusively in ethanol-withdrawn neurons. This neuroprotection could be explained by a decrease in NMDA-stimulated calcium influx after the administration of HU-210, found exclusively in ethanol-withdrawn neurons. By contrast, the inhibition of the cannabinoid system with the CB1 receptor antagonist rimonabant (SR141716) during ethanol withdrawal increased death of ethanol-withdrawn neurons without any modification of NMDA-stimulated calcium influx. Moreover, chronic administration of rimonabant increased NMDA-stimulated toxicity not only in withdrawn neurons, but also in control neurons. In summary, we show for the first time that the stimulation of the endocannabinoid system is protective against the hyperexcitability developed during alcohol withdrawal. By contrast, the blockade of the endocannabinoid system is highly counterproductive during alcohol withdrawal.

## Introduction

Continued excessive ethanol consumption can lead to the development of dependence that is associated with a withdrawal syndrome when ethanol consumption is interrupted or substantially reduced. This syndrome comprises psychological symptoms that contribute to distress and psychological discomfort, as well as physical signs that include tremor, agitation, delirium and in severe cases, convulsions and brain damages [1]. Neuroadaptive changes during ethanol consumption are believed to play an important role in the development of tolerance and physical dependence to ethanol. The alterations in glutamatergic transmission observed after ethanol exposure seem to play a key role in these responses, and could bring the brain to a hyperexcitable state [2].

Classical pharmacotherapies for treating alcohol-dependent subjects are addressed to reduce craving and early withdrawal symptoms (tremor, agitation, delirium), but they do not provide direct beneficial effects on the occurrence of brain damages, one of the major long-term consequences of alcohol dependence. Therefore, the search of novel compounds able to protect the brain against the degenerative events associated with alcohol dependence and withdrawal is a key objective, then concurring with the efforts for developing protective drugs for the treatment of acute or chronic neurodegenerative disorders. In this context, there is large evidence that cannabinoid agonists exert neuroprotection in several models of neuronal injury [3]. The mechanisms of this neuroprotection include, among others: (i) inhibition of excitatory glutamatergic transmission through presynaptic CB1 receptors [4]–[6]; and (ii) modulation of neuronal excitability exerted through the control of calcium (inhibition of voltage-dependent and other types of calcium channels) and potassium (activation of inwardly rectifying potassium channels) conductances [7]. These properties have been tested in multiple pathological conditions (e.g. hypoxia-ischemia, brain trauma, Parkinson's disease, Huntington's chorea), but never in an alcohol withdrawal situation. In this study, we wanted to test if cannabinoids could influence neurotoxicity during ethanol withdrawal. Besides, it has been described that CB1 receptor-deficient mice do not develop the changes in N-methyl-D-aspartate (NMDA) and γ-amino butyric acid (GABA)_A_ receptors observed in wild-type animals [8], suggesting that the endocannabinoid system may be implicated in the development of these glutamatergic and GABAergic neuroadaptations during chronic ethanol exposure. Accordingly, it would be of a great interest to examine whether the pharmacological activation or inhibition of the endocannabinoid system affects alcohol withdrawal-induced hypersensitivity to excitotoxic insults. To do this, we designed a series of experiments in an in vitro model of cultured murine cortical neurons to determine the changes in neuronal survival caused by the activation or the inhibition of the endocannabinoid signaling in conditions of chronic ethanol consumption and withdrawal. Experiments were conducted in basal conditions or after an excitotoxic stimulus with NMDA. In order to find the molecular bases of the effects found in the pharmacological experiments, we studied the changes in calcium influx and the expression of specific subunits of NMDA receptors.

## Results

### Ethanol withdrawal increases by 40% the sensitivity of neurons to excitotoxic injuries

**Figure 1:**
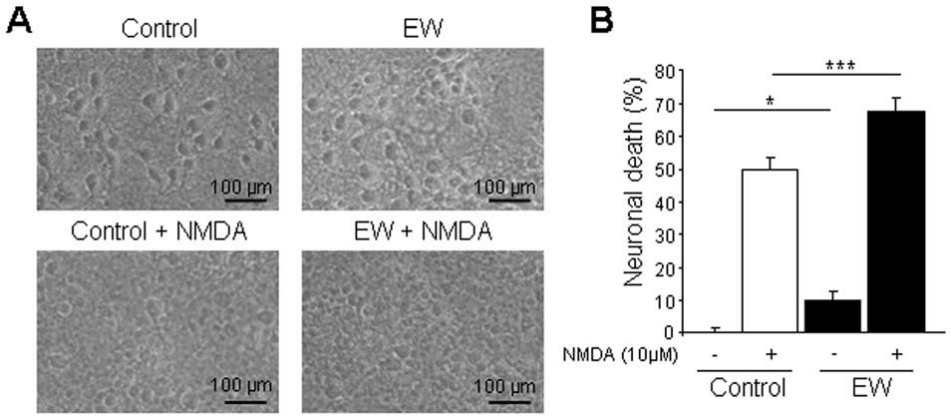
Ethanol withdrawal increases by 40% the sensitivity of neurons to excitotoxic injuries induced by NMDA (10 µM, 24 h) in cultured cortical neurons. (A) Phase-contrast photomicrographs show representative fields in the indicated conditions (Control, neurons not exposed to ethanol; EW, ethanol-withdrawn neurons). (B) Quantification of neuronal death. Values are means ± SEM (n = 17–20 wells/condition; N = 5 plates). Data were assessed by ANOVA test followed by Fisher's PLSD test (* *P*<0.05; *** *P*<0.001).

We developed an in vitro model of ethanol withdrawal which consisted in a chronic ethanol administration (100 mM, 3 days) and subsequent withdrawal (2 days), based on the model described by Nagy et al. [9]. Neuronal death was slightly increased in ethanol-withdrawn neurons (+10% cell death, *P*<0.05; figure 1B). In addition, we observed a significant increase in NMDA-stimulated cell death in ethanol-withdrawn neurons (+40% versus NMDA-treated control neurons; *P*<0.001; figure 1B) compared to NMDA-treated control (not exposed to ethanol) neurons, indicating that ethanol withdrawal renders neurons more sensitive to excitotoxic challenges. Representative phase-contrast photomicrographs under the indicated conditions are shown in figure 1A.

### Ethanol withdrawal decreases GluN2A subunit levels

In order to determine the origin of the increased sensitivity to NMDA-induced cell death in ethanol-withdrawn neurons, we examined the expression of the main NMDA receptor subunits, i.e., GluN1, GluN2A and GluN2B. GluN2A subunit expression, measured by quantitative real time RT-PCR (qRT-PCR), was significantly decreased in the withdrawal situation (*P*<0.05). No changes were observed for GluN1 or GluN2B subunit expression levels (table 1). Besides, we wanted to evaluate the possible changes after chronic ethanol administration in two important elements of the endocannabinoid system that have been related to alcohol addiction: CB1 receptors and fatty acid amide hydrolase (FAAH) levels [10]. CB1 receptor mRNA levels tended to increase after ethanol exposure and subsequent withdrawal, but this increase did not reach statistical significance (p = 0.098). The levels of FAAH enzyme remained unaffected.

**Table 1:** Ethanol withdrawal (EW) decreases GluN2A mRNA levels.

	CONTROL	EW
GluN1	100±15.5	75.5±11.2
GluN2A	100±12.8	56.2±6.2 *
GluN2B	100±15.2	73.9±13.3
CB1	100±28.5	205.4±45.5 a
FAAH	100±15.7	88.8±10.0

Levels of mRNA transcripts (measured by quantitative PCR) for the N-methyl-D-aspartate (NMDA) receptor subunits, CB1 receptors, and FAAH enzyme in cultured cortical neurons in control conditions (neurons not exposed to ethanol) or during ethanol withdrawal (EW group). Values are normalized to β-actin gene and expressed as percentages over control data (n = 3–4 samples/group; means ± SEM). Data were assessed by the Student's t-test (* *P*<0.05 vs. control; a, p = 0.097 vs. control).

**Figure 2:**
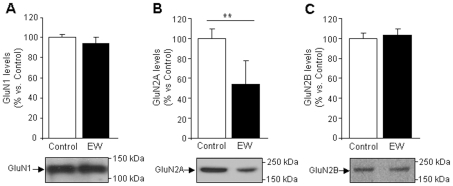
Ethanol withdrawal (EW) decreases GluN2A subunit levels. NMDA subunit protein levels measured by Western blot analysis: GluN2A levels (B) decrease during ethanol withdrawal, whereas the levels of GluN1 (A) and GluN2B (C) remain unaltered. Values are expressed as percentages over control data for each subunit and are means ± SEM (n = 3–5 samples/condition). Data were assessed by the t-Student's test (**P<0.01 vs. control).

To confirm the changes in mRNA expression and to investigate further the increase in neuronal death induced by ethanol withdrawal, we also performed western blot analyses of the NMDA receptor subunits (figure 2). Concordant with qRT-PCR results, GluN2A subunit levels decreased during ethanol withdrawal (figure 2B) (*P*<0.01), whereas GluN1 (figure 2A) and GluN2B (figure 2C) levels remained unaffected.

### The endocannabinoid system influences neuronal viability during ethanol withdrawal

**Figure 3:**
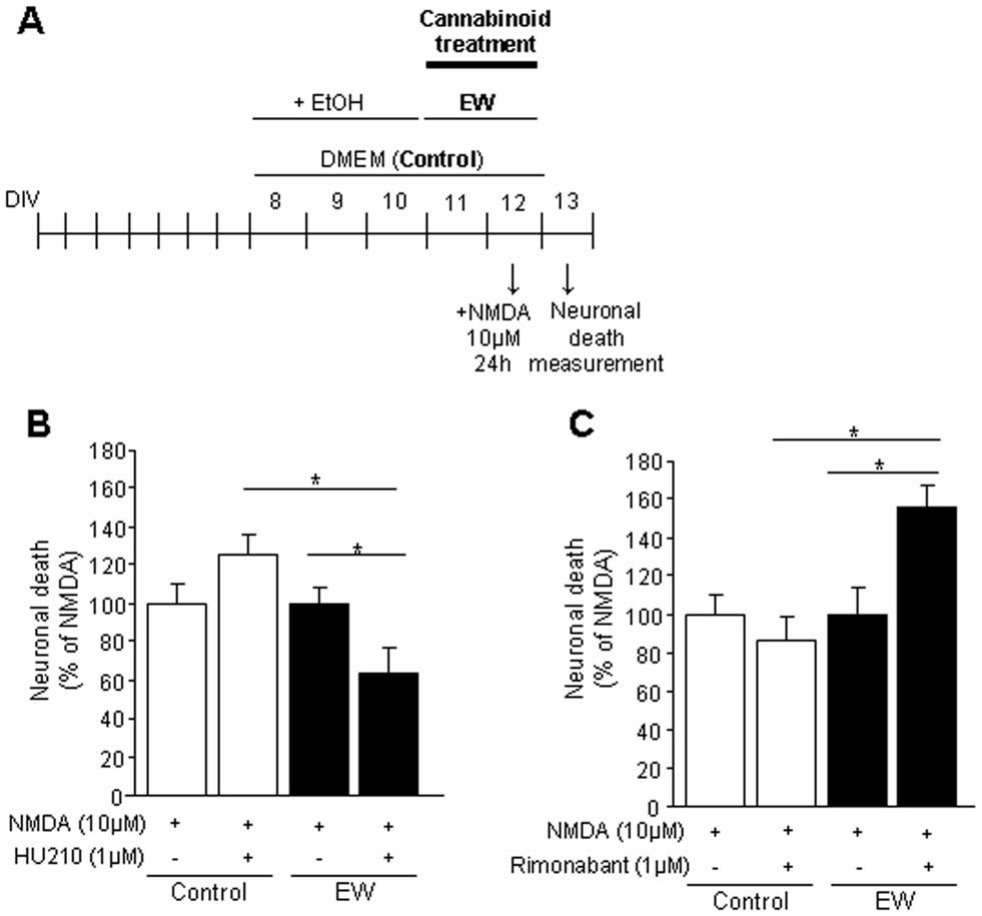
The acute manipulation of the endocannabinoid system influences neuronal viability during ethanol withdrawal. Effects of the acute activation or blockade of the endocannabinoid signaling on neuronal viability in control (neurons not exposed to ethanol) or ethanol–withdrawn neurons (EW). (A) Representative scheme of the experimental protocol. (B) The administration of the non-specific cannabinoid agonist HU-210 (1 µM) during ethanol withdrawal decreases NMDA-induced neuronal death in ethanol-withdrawn neurons. Interestingly, the neuroprotective effect of HU-210 is specific of alcohol withdrawal situation, since it has no effect on control neurons. (C) The acute administration of the CB1 antagonist rimonabant (1 µM) increases NMDA-induced neuronal death in alcohol-withdrawn neurons. Again, this effect is specific of the withdrawal situation and rimonabant, when administered acutely, has no effect on control condition. Values are normalized to NMDA and are means ± SEM (n = 14–18 wells/condition; N = 4 plates). Data were analyzed by the ANOVA test followed by the Fisher's PLSD test (* *P*<0.05).

**Figure 4:**
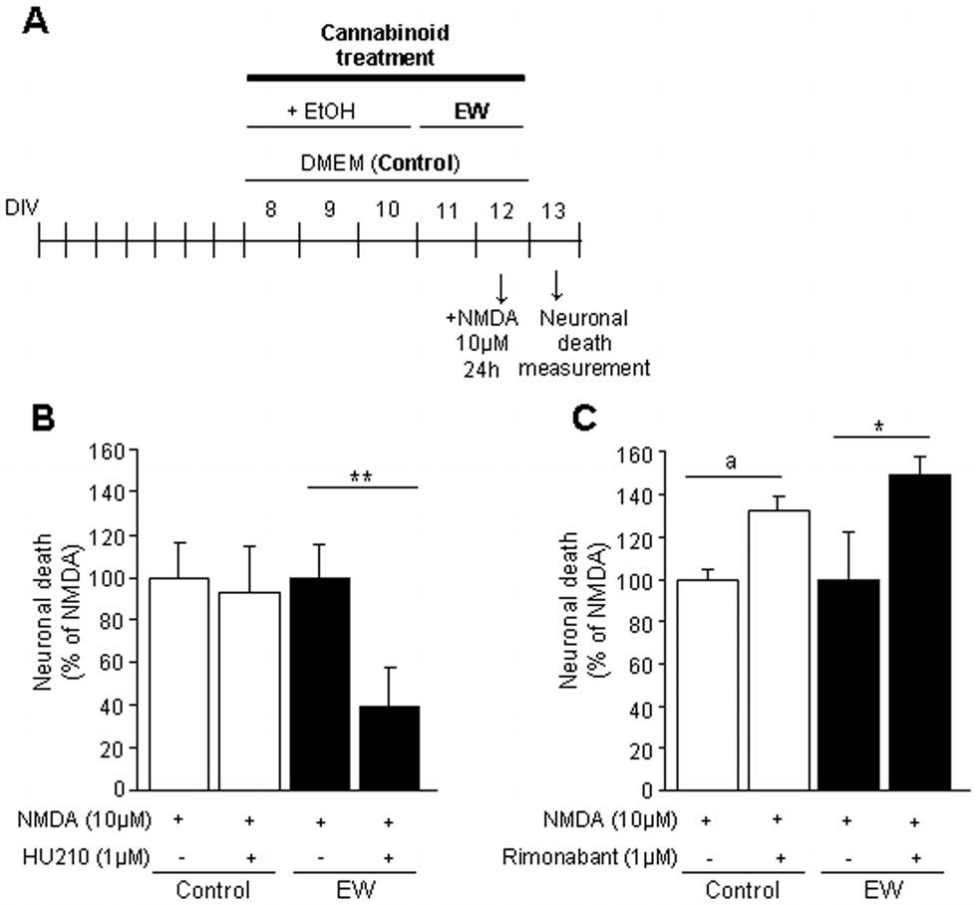
The chronic manipulation of the endocannabinoid system influences neuronal viability during ethanol withdrawal. Effects of the chronic activation or blockade of the endocannabinoid signaling on neuronal viability in control (neurons not exposed to ethanol) or ethanol–withdrawn neurons (EW). (A) Representative scheme of the experimental protocol. (B) The chronic administration of the non-specific cannabinoid agonist HU-210 (1 µM) decreases NMDA-induced neuronal death during ethanol withdrawal. The neuroprotective effect of HU-210 is specific of ethanol withdrawal situation, since it has no effect on the control condition. (C) The chronic administration of the CB1 antagonist rimonabant (1 µM) increases neuronal death. Moreover, the long-term administration of rimonabant tends to be neurotoxic not only for ethanol-withdrawn neurons but also for control neurons. Values are normalized to NMDA and are means ± SEM (n = 6–16 wells/condition; N = 3–4 plates). Data were analyzed by the ANOVA test followed by the Fisher's PLSD test. (*P<0.05; ** *P*<0.01; a, p = 0.0551).

We wanted to test the potential anti-excitotoxic effects of the cannabinoid agonist HU-210 in control or ethanol-withdrawn cultured cortical neurons. Schemes of the different treatments are shown in figures 3A and 4A. HU-210 was added to cell cultures acutely (only during ethanol withdrawal) (figure 3B) or chronically (both during the ethanol exposure period and during ethanol withdrawal) (figure 4B). In both cases, the addition of HU-210 induced a significant protection against NMDA-induced cell death (36% -*P*<0.05- and 61% -*P*<0.01- respectively). This effect was specific of the withdrawal situation since no neuroprotection by HU-210 was observed in control (not exposed to ethanol) neurons. By contrast, the administration of the CB1 antagonist rimonabant (both acutely -figure 3C- and chronically -figure 4C-) provoked a significant increase (+56% and +49% respectively, *P*<0.05) in NMDA-induced cell death in ethanol-withdrawn neurons. Moreover, the chronic administration of rimonabant to control (not exposed to ethanol) neurons also tended to increase NMDA-induced cell death (+33%; p = 0.0551) (figure 4C), thus suggesting that, in contrast to the effects of HU-210, the deleterious effects of rimonabant were also operative in cultured neurons that were never exposed to ethanol.

### The stimulation of the endocannabinoid system reduces NMDA-stimulated calcium influx in ethanol-withdrawn neurons

**Figure 5:**
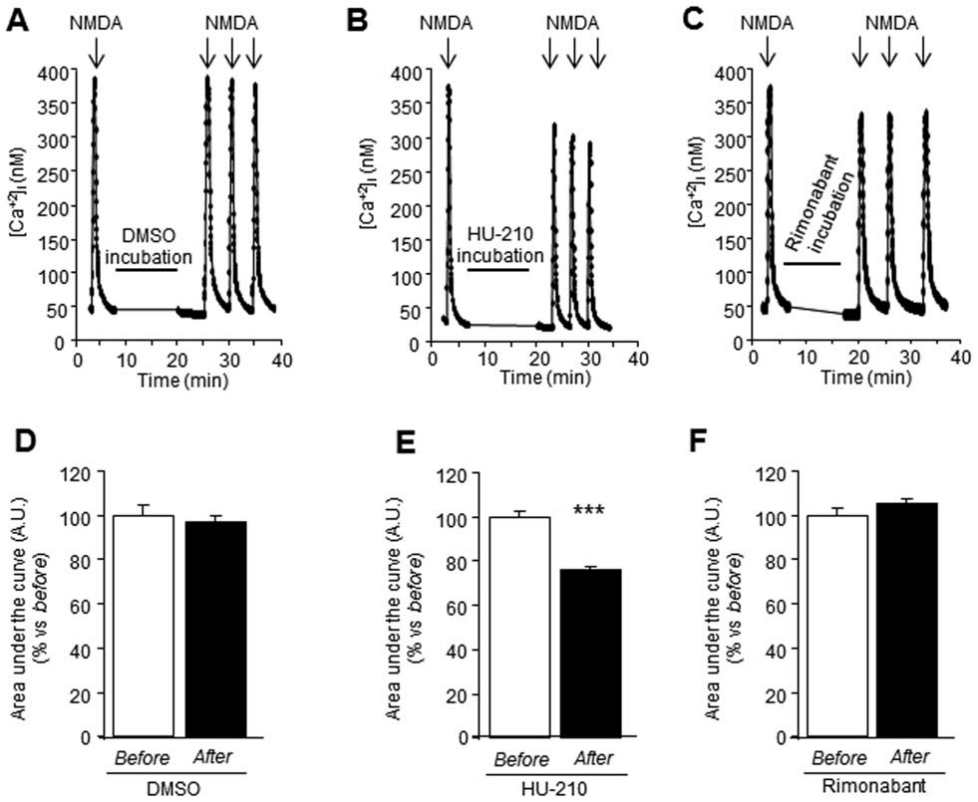
The stimulation of the endocannabinoid system reduces NMDA-stimulated calcium influx in ethanol-withdrawn neurons. (A, B, C) Representative recordings of single-cell calcium videomicroscopy for each experiment. A 30 sec exposure to NMDA (50 µM) produced a rapid calcium influx, which recovered over the following 2 min (*before*). (E) *After* the incubation (10 min) with the non-specific CB1 agonist HU-210 (1 µM), Ca^2+^ influx (induced by NMDA application) is significantly decreased in comparison to the stimulation *before*. Incubation (10 min) with DMSO (D) or rimonabant (1 µM) (F) does not modify NMDA-stimulated calcium influx. Values are expressed as % vs. *before* NMDA-stimulation ± SEM (N = 3–4; n (cell number) >60 cells). Data were analyzed by the t Student's test. (***P<0.001).

In order to explain the neuroprotective effect of the cannabinoid agonist HU-210 and the enhanced neurotoxicity found with rimonabant in ethanol-withdrawn neurons, we performed single cell calcium videomicroscopy analysis in ethanol-withdrawn neurons acutely incubated with vehicle (DMSO), HU-210 (1 µM) or rimonabant (1 µM). Representative curves of calcium influx for each experiment are shown in figures 5A, 5B and 5C. We observed a significant decrease (−24%; *P*<0.001) in NMDA-evoked Ca^2+^ influx after incubation with HU-210 (figure 5E) in ethanol-withdrawn neurons. This supports the idea that the beneficial effects on cell viability provoked by HU-210 could be related to a reduction of calcium entry through activated NMDA receptors, and a subsequent alleviation of calcium-dependent deleterious pathways. Incubation with vehicle (DMSO) (figure 5D) or rimonabant (figure 5F) did not produce any modification of calcium influx. In parallel, we performed the same experiments in sister control cultures (not exposed to ethanol). The administration of HU-210, rimonabant or vehicle to control (not exposed to ethanol) neurons did not have any effect on NMDA-induced Ca^2+^ influx (data not shown).

## Discussion

In the current study we show, first, that ethanol withdrawal increases both basal neuronal death and NMDA-stimulated neuronal death in primary cortical cultures, probably due to a change in the expression of NMDA receptor subunits. Second, we evidence for the first time that, during ethanol withdrawal, the stimulation of the endocannabinoid system protects neurons from excitotoxic insults, whereas its blockade aggravates neuronal death induced by NMDA. We show that the neuroprotective effect of HU-210 during ethanol withdrawal could be explained by a reduction in Ca^2+^ influx through NMDA receptors.

The increase in basal neuronal death found during alcohol withdrawal, which is concordant with previous results [11], suggests that chronic alcohol administration and subsequent withdrawal could decrease the threshold for triggering neurotoxic processes *per se*. This hypothesis is compatible with the enhanced brain damages observed in alcohol-dependent individuals who experienced successive periods of alcohol consumption, withdrawal and relapse [12], as well as in several in vivo experimental models of alcoholism [2]. In addition, we observed a significant increase in the sensitivity to NMDA toxicity in withdrawn neurons. This enhancement of excitotoxicity may reflect the neuronal adaptation to chronic ethanol exposure and could contribute to the generation of withdrawal symptoms such as seizures, pro-convulsive states and might also initiate excitotoxicity and loss of neurons in certain brain areas during alcohol withdrawal [12]. It is well accepted nowadays that changes in the glutamatergic system are one of the principal events responsible for the neuropathological alterations induced by chronic alcohol consumption in both laboratory animals and humans [2]. Our findings support the idea that NMDA receptor subunit composition changes after chronic alcohol administration and subsequent withdrawal, since we detected a decrease in GluN2A expression levels, whereas no changes were detected for GluN1 or GluN2B. It has been suggested that GluN2A-containing NMDA receptors promote neuronal survival and would exert a neuroprotective role against NMDA, while GluN2B-containing NMDA receptors would mediate neurotoxic processes [13]. If this hypothesis is correct, the decrease in GluN2A subunits induced by alcohol withdrawal would lead to an imbalance between the numbers of “trophic” versus “pro-neurotoxic” subunits and could explain the increase in neuronal death during ethanol withdrawal.

The implication of the endocannabinoid system in alcohol addiction has been largely studied in the last years. However, most studies have tried to reduce alcohol reinforcing effects or alcohol consumption [14], and less attention has been paid to the effects of cannabinoid stimulation or blockade on the neuropathological consequences of alcohol dependence and withdrawal. Our study shows that the stimulation of the endocannabinoid system protects ethanol-withdrawn neurons from excitotoxic cell death. This neuroprotection is present when the non-specific cannabinoid agonist HU-210 is administered both chronically (during ethanol administration and ethanol withdrawal) and acutely (only during ethanol withdrawal). Cannabinoid agonists have been proposed as neuroprotective molecules in several models of acute neuronal injury and chronic neurodegenerative diseases [15]. Among the proposed mechanisms of cannabinoid-receptor mediated neuroprotection, two are particularly relevant for the neurotoxicity caused by alcohol dependence and withdrawal: the inhibition of the presynaptic release of glutamate [16] and the inhibition of NMDA-induced Ca^2+^ influx [17]. It appears well-demonstrated that CB1 receptors are involved in these two neuroprotective properties of cannabinoid agonists and although HU-210 is a non-selective cannabinoid agonist, we assume that, in our experiments, HU-210 preferentially acts through the activation of CB1 receptors because -consistent with the suggestion that CB2 receptors are essentially present in glial cells [18] and considering the high purity of our neuronal cultures (thanks to the use of an anti-mitotic agent)- no mRNA encoding for CB2 receptors were detected in our cultures (data not shown).

As mentioned above, the neuroprotective effect of HU-210 against NMDA was exclusive of alcohol-withdrawn neurons, as it did not appear in control neurons (not exposed to alcohol). The absence of neuroprotection of HU-210 against NMDA excitotoxicity in cultured neurons has been previously reported [19], and the neuroprotective effect of HU-210 exclusively in ethanol-withdrawn neurons suggests that the neuroadaptive changes that occur during alcohol exposure sensitize cells to cannabinoid neuroprotective mechanisms, perhaps by enhancing CB1 receptor number and/or their signaling mechanisms. In agreement with this hypothesis, we found a 2-fold increase in the levels of CB1 receptor transcripts after alcohol cessation, which may explain the effects of HU-210 against NMDA excitotoxicity during alcohol withdrawal and its lack of efficacy in control neurons. To our knowledge, this is the first time that a neuroprotective role of HU-210 is found in conditions of alcohol withdrawal. In addition, we also found that the acute blockade of CB1 receptor-mediated signals during alcohol withdrawal with rimonabant increased NMDA-stimulated neuronal death, and again this neurotoxic effect was absent in control conditions. The data obtained with rimonabant also support the idea that the effects of HU-210 were CB1 receptor-mediated, as rimonabant is a selective CB1 receptor antagonist. The exacerbation of excitotoxin-induced neuronal death by inhibition of the cannabinoid receptors suggests that these neurons constitutively produce a cannabinoid receptor agonist. In accordance with this hypothesis, several authors have reported an increase in endocannabinoid production (anandamide and/or 2-arachidonoyl glycerol) after chronic alcohol exposure both *in vitro* and *in vivo* [20]–[24].

Excessive Ca^2+^ influx after the activation of NMDA receptors triggers neuronal death, whereas the suppression of the Ca^2+^ entry can protect cells from NMDA-induced cytotoxicity [25]. Given the inhibitory role played by CB1 receptors in Ca^2+^ homeostasis, the effects of CB1 receptor activation or blockade are likely followed by the corresponding reduction or increase in Ca^2+^ influx. Thus, we performed single cell calcium videomicroscopy analyses to determine the NMDA-evoked Ca^2+^ influx after the incubation with HU-210 or rimonabant. Consistent with its neuroprotective effect, the addition of HU-210 to alcohol-withdrawn neurons reduced NMDA-evoked Ca^2+^ influx. This effect was observed only in ethanol-withdrawn neurons, and not in control neurons (not exposed to ethanol; data not shown), in concordance with the results obtained in the excitotoxicity experiments. The addition of rimonabant to ethanol-withdrawn neurons, however, did not modify Ca^2+^ influx. Our data are supported by previous studies that described a modulation of NMDA-induced Ca^2+^ influx by CB1 agonists in different brain structures and through different mechanisms, although these studies were not conducted in conditions of alcohol withdrawal [17] [26].

In our study, the blockade of the endocannabinoid system with the CB1 antagonist rimonabant (both acutely and chronically) increased excitotoxic neuronal death in ethanol-withdrawn neurons. Moreover, the chronic blockade of the endocannabinoid system exacerbated excitotoxicity not only in ethanol-withdrawn neurons but also in control neurons (not exposed to ethanol). This suggests that the pharmacological blockade of the endocannabinoid transmission, especially during alcohol withdrawal, is highly counterproductive due to its ability to aggravate NMDA-stimulated cytotoxicity. This observation deserves further comments derived from its extrapolation to the in vivo situation. Increases in endocannabinoid levels have been described both in vivo and in vitro after acute [27] and more strikingly after chronic alcohol exposure [20] [22] [28], possibly in a cellular attempt to reduce damages due to alcohol [20] [22]. We can thus hypothesize that chronic CB1 receptor blockade, especially during alcohol exposure and withdrawal increases the sensibility to NMDA-induced cytotoxicity, which is concordant with the protective function assigned to the endocannabinoid generation in the above-mentioned studies. In any case, further experiments are needed to determine more precisely the cause of the deleterious effect of rimonabant during alcohol withdrawal.

Rimonabant has been proposed as a promising drug for the treatment of alcoholism, with positive effects on alcohol intake in laboratory animals under several administration protocols [29]. However, these preclinical data were not reproduced in the two clinical studies conducted to date with rimonabant (one in alcohol-dependent individuals and one in non treatment-seeking heavy alcohol drinkers) [30]–[31]. Moreover, further studies on the therapeutic potential of rimonabant in the treatment of alcohol dependence are apparently hindered, since the recent decision of the discontinuation of all clinical trials with this CB1 antagonist due to the occurrence of some psychiatric adverse effects. The results of the present work point in the same direction showing, for the first time, the potential neurotoxic effect of chronic administration of rimonabant in an *in vitro* model of alcohol withdrawal. It is also possible that the reason why rimonabant aggravated the deleterious effects of alcohol withdrawal in our in vitro model is related to its inverse agonist properties. If this is the case, it is likely that we will need the development of novel neutral antagonists, with no inverse agonist activity, that may be used efficaciously for treating alcohol craving with no additional neurotoxic effects.

In summary, these observations show, for the first time, that the stimulation of the endocannabinoid system could be protective against the hyperexcitability developed during alcohol withdrawal. By contrast, the blockade of the endocannabinoid system seems to be highly counterproductive during alcohol withdrawal due the ability of rimonabant to exacerbate NMDA-induced excitotoxicity.

## Materials and Methods

### Materials

NMDA and HU-210 were purchased from Tocris (Bristol, UK). Rimonabant was generously provided by Sanofi-Aventis (Montpellier, France). Dulbecco's modified Eagle's medium (DMEM), poly-D-lysine, laminin, glutamine, cytosine β-D-arabinoside, were purchased from Sigma-Aldrich (L'Isle d'Abeau, France).

### Ethics Statement

All animal procedures were performed in accordance with French (act no. 87-848; Ministère de l'Agriculture et de la Forêt) and European Communities Council Directives of November 24, 1986 (86/609/EEC) guidelines for the care and use of laboratory animals. The animal facility (agreement number: A14118015) and the experimenter (personal license number 14-65) were accredited by the Direction Départementale des Services Vétérinaires. Swiss mice were maintained on a 12∶12 h light/dark cycle, with ad libitum access to food and water. In agreement with the rules of humane killing on the day of use, pregnant mice were euthanized by anaesthetic overdose (carbon dioxide). Comité d'Ethique NOrmandie en Matière d'EXpérimentation Animale” (CENOMEXA) certifies that this study did not require referral to the regional ethics committee.

### Neuronal cultures

Neuronal cultures were prepared from Swiss mouse embryos (embryonic day 15–16) as described earlier [32]. Cortices were dissected and dissociated in DMEM, and plated on 24-well plates earlier coated with poly-D-lysine (0.1 mg/ml) and laminin (0.02 mg/ml). Cells were cultured in DMEM supplemented with 5% foetal bovine serum (Invitrogen, Cergy Pontoise, France), 5% horse serum (Invitrogen, Cergy Pontoise, France) and 2 mM glutamine. Cultures were maintained at 37°C in a humidified 5% CO_2_ atmosphere. Cytosine β-D-arabinoside (Ara C, 10 µM) was added after 3 days in vitro (DIV) to inhibit glial proliferation. All treatments were performed after 8 and/or 11–13 DIV as required.

### Ethanol and cannabinoid treatments

Following the model described by Nagy et al. [9], ethanol (100 mM in DMEM) was daily administered to cell cultures from DIV 8 during 3 consecutive days. No more ethanol was added to the culture cell medium afterwards to induce ethanol withdrawal (DIV 11–13). Cannabinoid compounds were dissolved in 100% dimethyl sulfoxide (DMSO) as a 10 mM stock and diluted with DMEM to their final concentrations. HU-210 (1 µM) or rimonabant (1 µM) were added to cell cultures chronically (from DIV 8 until DIV 13), or only during the ethanol withdrawal period (DIV 11–13). Excitotoxic challenges (see next section) were always performed at DIV 12 and neuronal death measured at DIV 13. Protein and RNA samples were extracted from separate series of plates at DIV 12.

### Induction of excitotoxicity and determination of cell viability

Excitotoxicity was induced by exposure to NMDA (10 µM) in serum-free DMEM supplemented with 10 µM of glycine for 24 h. This was always done at DIV 12 and neuronal death was quantified 24 h later (DIV 13) by measurement of the activity of lactate dehydrogenase (LDH) released from damaged cells into the bathing medium with a cytotoxicity detection kit (Roche Diagnostics, Mannheim, Germany). The LDH level corresponding to the maximal neuronal death was determined in sister cultures exposed to 200 mM NMDA (LDHmax). Background LDH levels were determined in sister cultures subjected to control washes (LDHmin). Experimental values were measured after subtracting LDHmin and then normalized to LDHmax–LDHmin to express the results as a percentage of neuronal death.

### Immunoblotting

Ice-cold TNT buffer (50 mM Tris-HCl pH 7.4; 150 mM NaCl; 0.5% Triton X-100)-dissociated cells were centrifuged (10,000 g, 4°C, 15 min), and protein content assessed by the BCA method (Pierce, France). Protein samples (20 µg) were resolved on a 10% SDS PAGE and transferred onto a polyvinylidene difluoride membrane. Membranes were blocked with 5% dried milk in Tris-buffered saline (TBS, 10 mM Tris; 200 mM NaCl; pH 7.4) containing 0.05% Tween-20 and incubated with primary antibodies (GluN1, GluN2A, GluN2B, 1∶200; all from Santa Cruz, Germany). After incubation with the corresponding secondary peroxidase conjugated antibody (1∶5000; Sigma Aldrich, France), proteins were visualized with an enhanced chemiluminescence ECL Plus immunoblotting detection system (Perkin Elmer-NEN, Paris, France).

### Quantitative real-time RT-PCR

Total RNAs were extracted from cultured cells by using the NucleoSpin RNA II kit from Macherey-Nagel, according to the manufacturer's instructions and eluted with RNase free water. One microgram of total RNAs from each sample was reverse transcribed using the Promega RT system (Promega, Charbonnieres, France; reverse transcription: 42°C for 1 h). Two primers were designed for each gene using the Beacon Designer software (Bio-Rad, Marnes-la-Coquette, France). Primer alignments studied with the BLAST database ensured the specificity of primers (see sequences for primers used in Table 2). PCR solutions were prepared with RNase-free water containing primers and IQ SYBR Green Supermix (Bio-Rad). For PCR amplification, 20 µl of mix were added to 5 µl of reverse transcription reaction diluted earlier (1∶20). Two negative controls were performed during each experiment. In the first control, we used samples without reverse transcription as a template to control contamination of RNA with genomic DNA. In the second control, we used RNase-free water instead of cDNA to prove that qPCR mixes were not contaminated with DNA. Assays were run in triplicate on the iCycler iQ real-time PCR detection system (Bio-Rad). The amplification conditions were as follows: Hot Goldstar enzyme activation, 95°C for 3 minutes; 50 cycles of PCR at 95°C, 15 sec and 60°C, 1 min. The levels of expression of interest gene were computed as follows: relative mRNA expression = 2^−(Ct of gene of interest)^ where Ct is the threshold cycle value.

**Table 2:** Sequences of primers used for quantitative PCR.

		forward	reverse
NMDA	GluN1	CTCTAGCCAGGTCTACGCTATCC	GACGGGGATTCTGTAGAAGCCA
	GluN2A	ACATCCACGTTCTTCCAGTTTGG	GACATGCCAGTCATAGTCCTGC
	GluN2B	CCAGAGTGAGAGATGGGATTGC	TGGGCTCAGGGATGAAACTGT
CB1		GTGTGCTGTTGCTGTTCATTGTG	CCTTGCCATCTTCTGAGGTGTG
FAAH		ATGAACCCGTGGAAGCCCTC	CGCCGATGTCAGTGCCTAAAC

### Single cell calcium videomicroscopy analysis

Primary cultures of cortical neurons (control -not exposed to alcohol- or ethanol-withdrawn) were loaded in the presence of HEPES-buffered saline solution containing 5 µM of fura-2/AM plus 0.1% pluronic F-127 (30 min, at 37°C; Molecular Probes, Leiden, the Netherlands) and incubated for an additional 30 min in HEPES-buffered saline solution. Experiments were performed at 22°C on the stage of a Nikon Eclipse inverted microscope equipped with a 75W Xenon lamp and a Nikon ×40, 1.3 numerical aperture epifluorescence oil-immersion objective. Fura-2 (excitation: 340 and 380 nm, emission: 510 nm) ratio images were acquired with a CCD camera (Princeton Instrument, Trenton, NJ, USA), and digitized using Metafluor 4.11 software (Universal Imaging Corporation, Cherter, PA, USA). Each experiment consisted in measuring NMDA-stimulated Ca^2+^ entry (25 µM NMDA) after 10 min incubation with HU-210 (1 µM), rimonabant (1 µM) or vehicle (DMSO). For each plate, basal NMDA-stimulated calcium entry was measured prior to incubations.

### Statistical analyses

All data were assessed by Student's *t*-test or one-way analysis of variance, as required. In the case of using analysis of variance, we used the Fisher's PLSD test as post-hoc test. All statistical analyses were done using the Statview software.
